# An auditory-responsive interneuron descending from the cricket brain: a new element in the auditory pathway

**DOI:** 10.1007/s00359-022-01577-8

**Published:** 2022-10-08

**Authors:** Stephen M. Rogers, Konstantinos Kostarakos, Berthold Hedwig

**Affiliations:** 1 Department of Zoology, University of Cambridge, Cambridge, CB2 3EJ UK; 2 Department of Life Sciences, University of Lincoln, Brayford Pool Campus, Lincoln, LN6 7TS UK; 3 Institute of Biology, University of Graz, Graz, Austria

**Keywords:** Cricket acoustic communication, Auditory processing, Descending interneuron, Sensory motor integration

## Abstract

Crickets receive auditory information from their environment via ears located on the front legs. Ascending interneurons forward auditory activity to the brain, which houses a pattern recognition network for phonotaxis to conspecific calling songs and which controls negative phonotaxis to high-frequency sound pulses. Descending brain neurons, however, which are clearly involved in controlling these behaviors, have not yet been identified. We describe a descending auditory-responsive brain neuron with an arborization pattern that coincides with the ring-like auditory neuropil in the brain formed by the axonal arborizations of ascending and local interneurons, indicating its close link to auditory processing. Spiking activity of this interneuron occurs with a short latency to calling song patterns and the neuron copies the sound pulse pattern. The neuron preferentially responds to short sound pulses, but its activity appears to be independent of the calling song pattern recognition process. It also receives a weaker synaptic input in response to high-frequency pulses, which may contribute to its short latency spiking responses. This interneuron could be a crucial part in the auditory-to-motor transformation of the nervous system and contribute to the motor control of cricket auditory behavior.

## Introduction

Insect brains integrate multimodal sensory activity from the principal sense organs of the head, the compound eyes, ocelli and antennae, which encompass visual, chemosensory and mechanosensory information. Their brains also decide upon and initiate motor programs in response to complex sensory processing (Barron et al. 2015; Cheong et al. 2020) and the internal motivational state of the animal (Herberholz and Marquart 2012; Mowrey and Portman 2012). Because of the central importance of this executive role, sensory information from sense organs that are not located on the head is also often conveyed to the brain. In Orthopteran insects, this includes two major sensory systems crucial to their lifestyle. One is the cercal air-current-sensitive escape pathway (Edwards and Palka 1974) with giant interneurons projecting from the terminal ganglion to the brain (Hirota et al. 1993; Yamao et al. 2022) which controls the escape response (Oe and Ogawa 2013; Sato et al. 2021). The other sensory modality is the auditory pathway underlying acoustic communication. Auditory interneurons ascend to the brain from an auditory neuropil either in the metathoracic ganglion (grasshoppers, the Caelifera) or in the prothoracic ganglion (crickets/bushcrickets, the Ensifera), (Stumpner and Helversen 2001; Hedwig and Stumpner 2016). Even though the thoracic ganglia, which are the primary destination of auditory sensory information, also produce the motor patterns that underlie auditory-mediated behavior, the brain is essential for species-specific pattern recognition and for the decision to initiate appropriate auditory motor responses by activating descending pathways.

Auditory-mediated behaviors in crickets fall into two broad classes: avoidance or escape behaviors in response to the high-frequency echolocating calls of bats, i.e., negative phonotaxis; and mate-finding where female crickets orientate and walk or fly towards males producing species-specific song patterns, i.e., positive phonotaxis (Wyttenbach and Hoy 1996).

For the bat avoidance behavior, interneuron AN2, which receives monosynaptic inputs from auditory afferents in the prothoracic ganglion and ascends to the brain (Rheinlaender et al. 1976; Wohlers and Huber 1982; Hennig 1988; Hardt and Watson 1994), is sufficient to elicit an avoidance steering response (Nolen and Hoy 1984). The neuron has broad frequency tuning and responds vigorously to high-frequency sound pulses; it sends this information up to the anterior lateral protocerebrum of the brain, which in flying crickets is required to trigger the steering response (Nolen and Hoy 1984).

Whereas bat avoidance can simply be based on the carrier frequency of the sound pulses, positive phonotaxis to conspecific songs requires pattern recognition, prior to any motor responses. In *Gryllus bimaculatus* interneuron AN1, which also receives monosynaptic input from auditory afferents, has a narrow frequency tuning centered on the calling song frequency of 4.8 kHz (Wohlers and Huber 1982; Schildberger 1984; Hardt and Watson 1994). AN1 ascends to the brain and closely copies the pulse pattern of the calling song. As the species-specific songs are complex, the receiving cricket must not only recognize the correct carrier frequency but also determine if the song conforms to the species-specific pattern of sound pulses and intervening silent pulse intervals. A pattern recognition network tuned towards the species-specific pulse rate of the calling song has been identified in the brain (Kostarakos and Hedwig 2012; Schöneich et al. 2015; Clemens et al. 2021).

Some descending interneurons which project from the brain to the thoracic ganglia show auditory responses (Boyan and Williams 1981; Staudacher and Schildberger 1998; Staudacher 2001; Zorovic and Hedwig 2013). These neurons, however, require high-intensity sound pulses to respond, and do not show the reliable auditory responses that would be needed to robustly support ongoing auditory-related motor activity. Therefore, for bat avoidance steering as well as for positive phonotaxis, the auditory-to-motor interface, a crucial part in the organization of the auditory behavior, is barely understood. Here, we add to our understanding of the neural organization of the auditory pathway in crickets and describe an interneuron, that robustly carries forward auditory signals from the brain to the posterior ganglia. The neuron originates in the protocerebrum from the ring-like auditory neuropil formed by the axons of auditory ascending neurons and local neurons (Kostarakos and Hedwig 2012) and reliably responds to acoustic stimuli.

## Materials and methods

### Experimental animals

Female bispotted field crickets (*Gryllus bimaculatus*) were taken from a colony at the Department of Zoology Cambridge, kept at 26–28 °C, and a 12 h–12 h light: dark cycle. Animals had free access to fish food flakes, muesli and water. Adult females seven–fourteen days old were selected for the recordings and were checked for intact tympanal membranes. Experiments complied with the principles of Laboratory Animal Care (ASAB Ethics Committee and ABS Animal Care Committee 2021).

### Intracellular recording

Animals were mounted vertically with their heads pushed through a rubber gasket formed from the finger of a latex glove mounted on a horizontal plastic platform onto which the head was fixed into position with a beeswax and rosin mix. The head capsule was opened frontally, and the brain was exposed. It was mechanically stabilized with a platform placed under its dorsal side and a ring made from fine (50 µm diameter) tungsten wire placed gently on its ventral surface. At all times, the brain was covered with insect saline (either: in mM: NaCl 140; KCl 10; CaCl_2_ 7; NaHCO_3_ 8; MgCl_2_ 1; *N*-trismethyl-2-aminoethanesulfonic acid 5; *D*-trehalose dehydrate 4, pH 7.4, or the same except with NaHCO_3_ 4, HEPES 10 replacing *N*-tris methyl-2-aminoethane sulfonic acid 5 and no *D*-trehalose).

Intracellular recordings from brain neurons were obtained with sharp microelectrodes pulled (DMZ Universal Puller, Zeitz-Instruments, Martinsried, Germany) from thin-wall borosilicate glass capillaries (BF-100-78-10, WPI, Hertfordshire, UK). The tip of electrodes was filled either with 0.25% Alexa 555 or 5% Lucifer yellow in 0.5 M LiCl (Sigma-Aldrich, St Louis, MO, USA), the shaft was backfilled with 1 M lithium chloride. They had resistances of 40–50 MΩ when the tip was filled with Alexa 555, or 70–100 MΩ when the tip was filled with Lucifer yellow. Signals were amplified with a DC amplifier with current injection facility (BA-01X, NPI, Tamm, Germany). The fluorescent dye was iontophoretically injected into recorded neurons by a constant hyperpolarizing current of 0.5 nA. Neurons were recorded independently by two researchers. Three recordings and stainings were obtained from B-DARN1 in different crickets: one recording (1) lasted 30.8 min and was obtained from the descending axon, outside the ring-like neuropil. This recording provided most of the data used in this paper. Another recording (2) provided detailed structure and physiological responses; the third recording (3) lasted only briefly but revealed the major response characteristics and main morphological features of the neuron.

### Acoustic stimulation

Acoustic stimuli were simultaneously presented via two speakers (Neo 13S, Sinus Live, Conrad Electronics, Hirschau, Germany) positioned at the left and right side at 36° to the cricket’s thorax at a distance of 22.2 cm unless otherwise stated. Auditory stimuli were created with the software Cool Edit 2000 (Syntrillium, Phoenix, USA), with a sampling rate of 44.1 kHz, a 16-bit amplitude resolution. Sound intensities for each speaker were individually adjusted one at a time to 75 dB SPL relative to 10^–5^ N m^−2^ at the position of the cricket and were measured with a Bruel and Kjaer free field microphone (type 4191) connected to a measuring amplifier (type 2610). When both speakers were simultaneously active, the sound intensity was 3 dB greater. The fine-scale homogeneity of the sound field could not be checked and may have been influenced by the electrophysiological set up. Calling songs at 65 and 55 dB SPL were produced by adjusting the output using Cool Edit. A 3-s courtship song sample was made from a recording of a live cricket and played through a continuous loop using CoolEdit.

### Neuron morphology

After injection of the fluorescent dye, the brain and SOG were removed, fixed in 4% paraformaldehyde for 30 min and dehydrated using an EOH series. The tissue was finally cleared in methyl salicylate. Wholemount pictures of the brain were taken with an epifluorescence microscope (Axiophot, Carl Zeiss, Wetzlar, Germany) with emission/absorption filters matching the spectral properties of Lucifer yellow or Alexa 555. The brains were also imaged on a Leica SP5 confocal microscope (Wetzlar, Germany). Neuronal arborizations in the brain were reconstructed both manually from the photographs taken on the Axiophot microscope and drawing the neuron using CorelDraw X17, and from maximum intensity projections taken from confocal image stacks using ImageJ Fiji (http://imagej.nih.gov/ij). The neuron was anatomically characterized in 3 specimens.

### Data analysis

The envelope of sound stimuli and the neuronal signal were sampled at 40 kHz per channel (Micro1401 mk II, CED, Cambridge, UK) to the hard disk of a PC. Data analysis was performed offline with Spike II (CED, Cambridge, UK). Peri-stimulus time histograms and waveform averages were produced using Spike II. Statistical tests and graphs were produced using R (version, 3.6.3, the R foundation for statistical computing); NCSS 10 Statistical Software (2015, Kaysville, Utah, USA), and Sigmaplot 11 (Systat, Inpixon, Düsseldorf, Germany).

## Results

### Anatomy

**Fig. 1:**
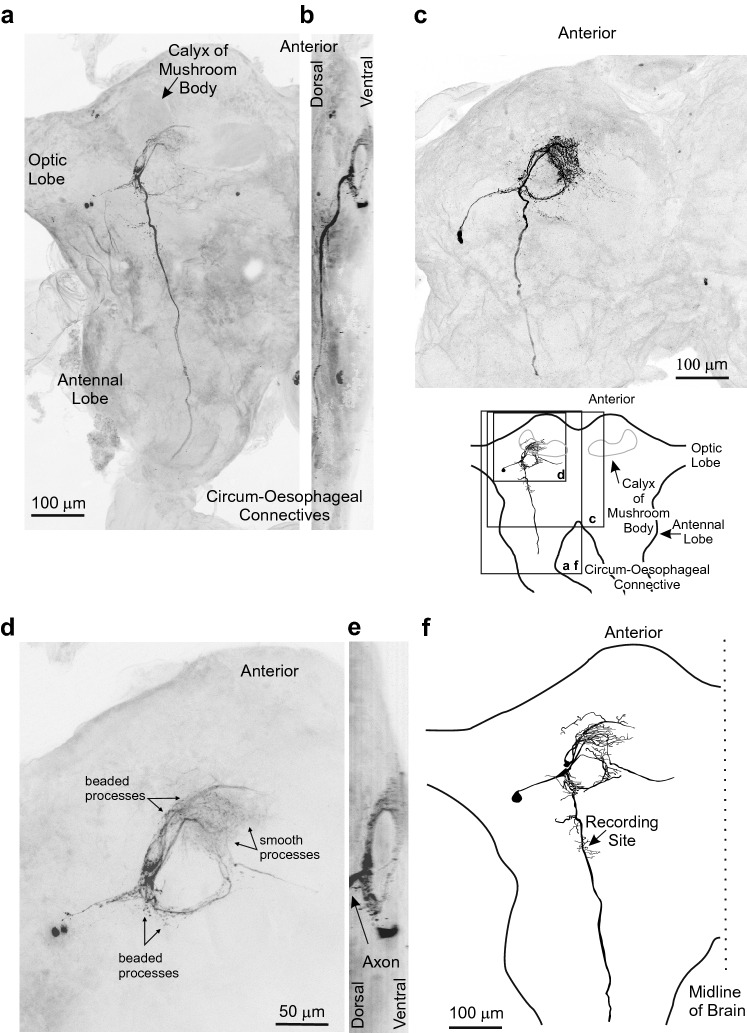
Anatomy of the Brain Descending Auditory-Responsive Neuron (B-DARN1). **a** Maximum intensity projection view of B-DARN1, recorded outside the ring-like neuropil, from a confocal imaging stack in ventral view and **b** lateral view (data from recording 1) **c** B-DARN1 recorded at the transition between the axon and the ring-like neuropil in ventral view (data from recording 2). **d** The ring-like arborization of B-DARN1 from recording 1 in higher magnification in ventral view and **e** lateral view. The axon projects dorsally from the ring and is not apparent in **d** but is labeled in **e**. **f** Drawing of the anatomy of B-DARN1 from recording 1 based on an image stack taken on a Zeiss Axiophot epifluorescence microscope. The recording site is indicated. The inset shows the locations of **a**–**f** in relation to an outline of a cricket brain with labeled landmarks in ventral view

The Brain Descending Auditory-Responsive Neuron (B-DARN1) shows a prominent ring-like arborization in the ventral protocerebrum, completely distinct and ventral to the calyx of the mushroom body (Fig. 1). This ring-like arborization is an anatomical feature of several brain auditory interneurons, including the axonal arborizations of the ascending interneurons AN1 and AN2 (Wohlers and Huber 1982), which provide the principal auditory input to the brain, and at least several local sound pattern recognition brain neurons such as LNs 1–5 (Kostarakos and Hedwig 2012; Schöneich et al. 2015).

The most anterior and finely branched part of the ring arborization of B-DARN1 (Fig. 1a, c, d) lies just under the ventral surface of the brain (Fig. 1b, e). Larger branches posterior to this fine arborization form the enclosed ring-like structure (Fig. 1a, c, d). An axon projects initially dorsally out of the ring arborization (Fig. 1b, e) before running posteriorly in a dorsal region of the brain towards the circum-oesophageal connectives (Fig. 1a–c). In all recordings, the injected dyes faded before, or just after, reaching the circum-oesophageal connective and it was not possible to reveal the neuron’s output regions. Within the ring arborization, the smaller branches present a mixture of smooth and beaded processes (Fig. 1d). The heaviest concentration of beaded processes appeared to be in the lateral part of the ring near where the axon arises, whereas the medial neurites ended in a mass of fine smooth branches (arrows Fig. 1d). The arborization pattern of B-DARN1 closely resembles that of the axonal arborization pattern of the ascending auditory interneuron AN1, except that stains of B-DARN1 revealed a lateral process that ended in somata in the lateral protocerebrum posterior to the stalk of the optic lobe, in a region occupied by the somata of several other auditory interneurons (Kostarakos and Hedwig 2012). Two of the three stains of B-DARN1 (recordings 1 and 2) indicate possible of two adjacent cell bodies, perhaps indicative of two dye-coupled neurons. There appear to be two somata in the stain from recording 1 (Fig. 1a, d) but a closer inspection of the image data revealed that the more medial spot is diffuse, pallid, and very superficial to the surface of the brain and had no primary neurite. This spot was not apparent in the image stack taken on the Zeiss Axiophot fluorescence microscope used to make the drawn reconstruction of the neuron (Fig. 1f). In Fig. 1c, made from recording 2, there may be two primary neurites, but what appears to be a pair of cell bodies may be a single waisted cell. Most of the anatomical data presented here (Fig. 1a, b, d–f) were made from a recording in which the electrode was placed well away from the ring arborization (arrow Fig. 1f).

### Response to natural chirp-like song patterns

**Fig. 2:**
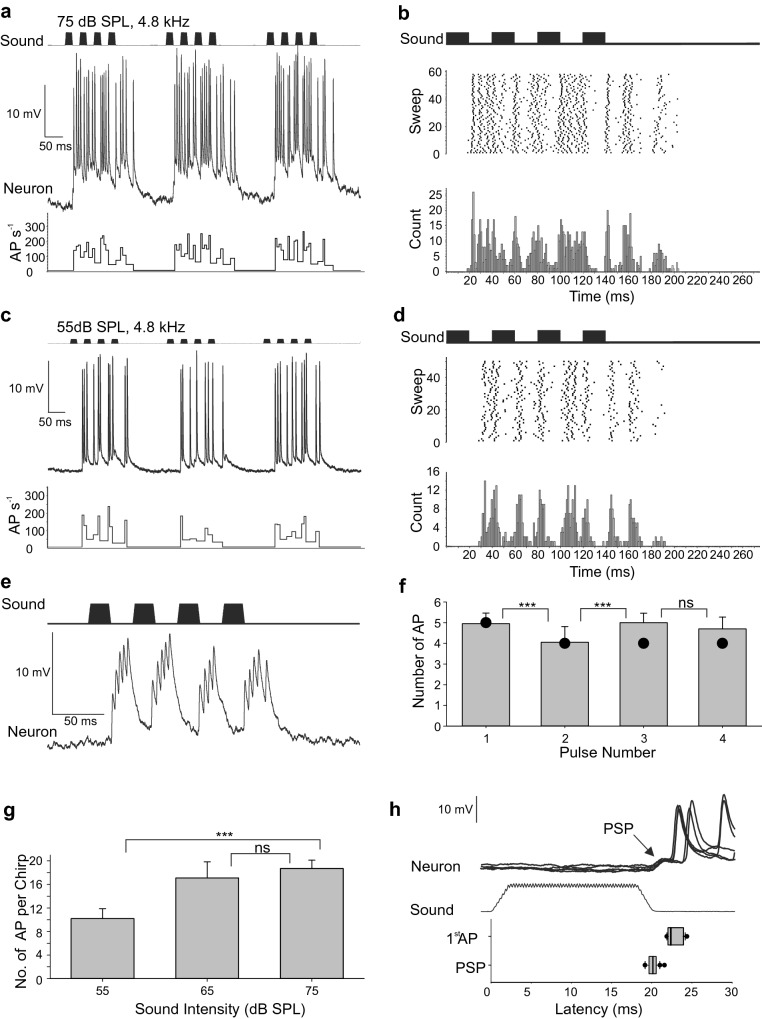
Response of B-DARN1 to binaural stimulation by natural chirp-like song patterns. All data are from recording 1 apart part e, which is from recording 2. **a** The sound envelope (top trace) represents three successive chirps at 75 dB SPL. Each chirp consists of four 20 ms pulses at 4.8 kHz separated by 20 ms silent intervals, with a chirp interval of 140 ms. B-DARN1 activity (middle trace) and its instantaneous spike rate (bottom trace). **b** PST histogram and raster plot of the AP response of B-DARN1 to 58 successive chirps at 75 dB SPL. **c**, **d** The response of B-DARN1 and histograms and raster plots of the spike response to the chirps at 55 dB SPL. **e** Response of B-DARN1 to a chirp-like sound pattern at 4.8 kHz and 75 dB SPL in recording 2. **f** Mean number of action potentials (± SD) in a chirp evoked by each sound pulse. The dots show the number of AP evoked by the 58th chirp. **g** Mean number of AP (± SD) evoked by chirps played at 55, 65 and 75 dB SPL. **h** Five successive overlaid responses of B-DARN1 triggered by the onset of a chirp showing the post-synaptic potential (PSP) that precedes the onset of spiking. The box-plot shows the medians (line), interquartile ranges (boxes) and 90% data range (whiskers) of the latencies of the initial PSP and first AP of the response (*N* = 20). The graph is at the same time scale and aligned on the recording traces

The response of B-DARN1 was tested to song patterns modeled on natural *G. bimaculatus* chirps, consisting of four 20 ms pulses at 4.8 kHz separated by 20 ms silent pulse intervals with a chirp interval of 140 ms (chirp rate 3.57 s^−1^). B-DARN1 responded vigorously to binaural stimulation by calling song (Fig. 2), with bursts of action potentials (AP) following each sound pulse, which surmounted sustained depolarizations with little repolarization during pulse intervals in one of the recordings (Fig. 2a–d). The spiking response followed the chirp pattern, but not as tightly as in the ascending auditory interneuron AN1 (Wohlers and Huber 1982; Hardt and Watson 1994; Kostarakos and Hedwig 2012), and the pulse intervals were difficult to discern in stimulus histograms or raster plots (Fig. 2b, d). At 75 dB SPL, the first sound pulse led to a burst of five nearly evenly spaced AP approximately every 6 ms. The response to the subsequent pulses was more complex and biphasic. It consisted of an initial AP approximately 22 ms after the onset of a sound pulse followed by a gap of about 10 ms in which few AP occurred, but after which several further AP were evoked (Fig. 2b). The response was terminated by 1–2 AP occurring approximately 40 ms after the end of the last pulse in a chirp. This biphasic pattern was particularly clear in the less intense response to songs played at 55 dB SPL (Fig. 2c), in which seven distinct peaks occurred in the raster plot and histogram (Fig. 2d). A single AP occurred sporadically > 40 ms after the last pulse in approximately 17% of chirps. In recording 2 of B-DARN1 (Fig. 2e), the membrane potential repolarized after each response to a pulse and the pulse structure could be clearly discerned in the AP response.

At 75 dB SPL, the number of APs per pulse was consistent across repeated stimulation, a latency of 20 ms was used as a cut-off to isolate the response from successive pulses (Fig. 2f; see below). 75% of first pulses evoked 5 AP, 60% of second pulses evoked 4 AP, 80% of third pulses evoked 5 AP, and 60% of fourth pulses evoked 5 AP, with all other AP counts being ± 1 of these modal values (*N* = 20). The decrease in AP count between the first and second pulses was significant (Fisher’s exact test, *P* = 4 × 10^–4^), as was the subsequent increase again in the third pulse (Fisher’s exact test, *P* = 9 × 10^–5^). Even after 58 repeated chirps, the numbers of evoked APs were 5, 4, 4, 4 for each pulse of a chirp, indicating that B-DARN1 copied sound patterns with high fidelity for extended periods (circles, Fig. 2f).

Decreasing the sound intensity of chirps to 65 dB SPL from 75 dB SPL led to a modest but non-significant decrease in response (Fig. 2g), evoking 16.6 ± 1.8 AP per chirp as compared to 18.5 ± 1.0 (Mann–Whitney test, *N* = 20, *Z* = 1.88, *P* = 0.060). A further reduction to 55 dB SPL (Fig. 2c, d, g) led to a significant decrease in the response, with only 10.1 ± 1.7 AP per chirp (Mann–Whitney test, *N* = 20, *Z* = 5.32, *P* < 1 × 10^–7^).

The spiking response was preceded by a Post-Synaptic Potential (PSP), with an amplitude of 3.2 ± 0.3 mV which occurred 20.1 ± 0.6 ms (N = 20, means ± SD) after the onset of sound and showed little temporal variation between stimuli (Fig. 2h). This clearly discernible PSP and the depolarizations underlying its spiking identifies this neuron as physiologically distinct from AN1, since only axonal AP can be seen in brain recordings of AN1. The latency of B-DARN1 spikes was more variable, occurring 22.8 ± 0.9 ms after the onset of sound, but the box-plot (Fig. 2h) suggests a more asymmetric distribution, with some AP delayed for a longer period (median time of first AP 22.4 ms, interquartile range 22–23.9 ms). In approximately one in twenty chirps, the initial PSP supported an AP. In recording 2, the initial depolarization directly produced an AP without a clearly discernible PSP, and subsequent AP in the bursts were supported upon a sustained depolarization (Fig. 2e).

### Response to changes in pulse duration

The effect of pulse duration was investigated by playing songs in which the pulse durations of the four-pulse chirps were systematically varied from 5 to 100 ms, while the pulse interval was kept at 20 ms, and the chirp interval at 140 ms. Trains of 12 chirps were played of each chirp type, separated by 2 s silent intervals. Song patterns were presented in pseudo-random order so that long- and short-pulse duration chirp types alternated. The first response of a new stimulus type was excluded from analysis since this response was typically much greater (up to 30%) following a recovery in the silent period between chirp trains.

**Fig. 3:**
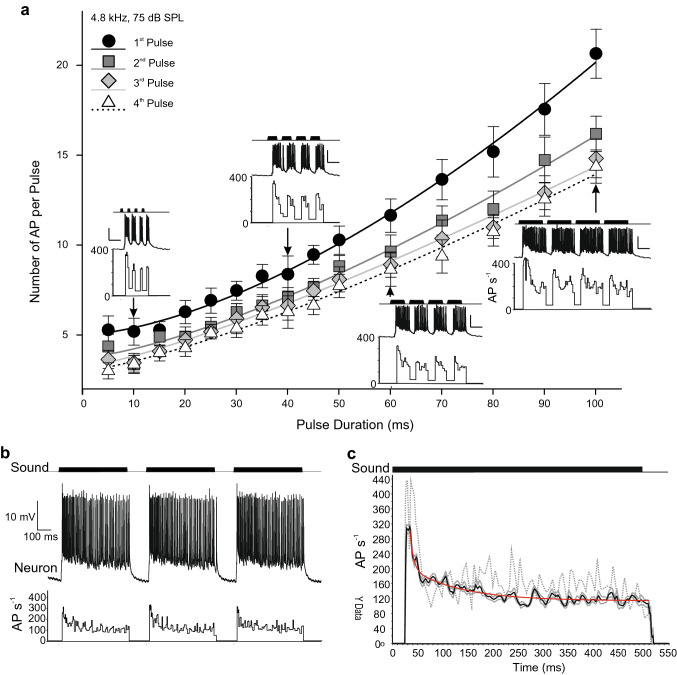
Effect of pulse duration on B-DARN1 response using four-pulse chirps with 20 ms pulse intervals. All sounds were at 4.8 kHz and 75 dB SPL; data are from recording 1. **a** The mean numbers of APs (± SD) evoked by chirps with different pulse durations for each of the four successive pulses within the chirp (*N* = 11). The data are fitted with lines of the form *y* = *y*_0_ + *ax*^*b*^, see Table 1 for details. B-DARN1 responses to chirps of different pulse durations are shown on the graph at the pulse durations indicated. Diagrams show the sound stimulus (top) the neuronal responses (middle) and the instantaneous AP firing rates (bottom). Scale bars are 10 mV and 50 ms. **b** Response of B-DARN1 to three successive 500 ms sound pulses at 4.8 kHz separated by 140 ms intervals, with sound stimulus (top) and the instantaneous firing rate (bottom). **c** The mean instantaneous firing rate (black line) ± SEM (solid gray lines) of B-DARN1 during 500 ms sound stimuli (*N* = 9). The dotted line shows the initial dis-adapted response to this stimulus after a 5-s silent interval. There were 67 ± 5.4 AP per stimulus. The red line shows a fitted double exponential decay function illustrating the adaption of the neuron. Data are from recording 1

The spiking activity in B-DARN1 reflected the pulse duration within chirps (Fig. 3). The strongest responses were to the first pulse within a chirp (black symbols, *N* = 11, Fig. 3a), and the number of evoked AP decreased in each successive pulse (gray to white symbols, Fig. 3a). Pulses shorter than 20 ms evoked similar numbers of AP, suggesting a strong phasic initial response. As pulse duration increased, so did the number of evoked AP. Between 20 and 50 ms, there was a rising curve, consistent with an ongoing strong phasic response, but for the longest pulse lengths, the relationship became approximately linear, suggesting a shift to a tonic response. The data could be fitted with a non-linear regression of the form *y*_0_ + *ax*^*b*^, with *R*^2^*s* of 0.95–0.96 (Table 1), where y_0_ is the predicted minimum number of AP that can be evoked and *ax*^*b*^ describes the phaso-tonic character of the response. The y_0_ values suggest a minimum burst of 5 AP for the first sound pulse, decreasing to 3 AP for the fourth (Table 1). The constant “a” ranged from 0.01 to 0.04, while the exponent “*b*” ranged from 1.58 to 1.25 across the four pulses of a chirp (Table 1). This relationship suggests an initial strong phasic response for pulses up to 50 ms duration which gave way to a tonic response in longer pulses which evoke proportionately fewer AP per ms of sound pulse.

**Table 1:** Estimates and significances of non-linear regressions of the form *y* = *y*_0_ + *ax*^*b*^ fitted to the number of evoked action potentials (*y*) in relation to sound pulse duration (*x*)

Pulse	*Y*_0_	*a*	*b*	*R*^2^	*F*_2,162_	*P*
1	5.0 ± 0.19	0.01 ± 0.003	1.58 ± 0.07	0.96	1970.1	8.2 × 10^–116^
2	3.7 ± 0.18	0.02 ± 0.006	1.40 ± 0.06	0.96	1818.8	4.4 × 10^–113^
3	3.2 ± 0.19	0.04 ± 0.010	1.25 ± 0.06	0.95	1668.1	3.8 × 10^–110^
4	2.99 ± 0.17	0.03 ± 0.077	1.31 ± 0.06	0.95	1730.5	2.2 × 10^–111^

See Fig. 3A

The phaso-tonic characteristics of B-DARN1 were revealed in its response to sound stimuli consisting of 500 ms long sound pulses separated by 140 ms silent intervals (Fig. 3b). The dis-adapted response to the first stimulus in the series, which occurred after a 5 s silent interval, had a firing rate 37.5% higher during the initial phasic response, and 33% higher during the tonic part of the response (dotted line Fig. 3c) than the averaged response to the nine subsequent repeats of the stimulus (Fig. 3c; mean is shown as a solid black line and the SEM as gray lines). The peak spike rate occurred between 28 and 34 ms after the onset of the sound pulse when B-DARN1 fired at 322 ± 16 AP s^−1^, but activity halved over the subsequent 60 ms, and then decreased more slowly as the stimulus persisted, reaching a tonic firing rate after approximately 225 ms, which by the end of the stimulus was 106 ± 8 AP s^−1^. The adaptation of the response could be closely fitted with a double exponential decay function of the form *f* = *y*_0_ + _*ae*_^(−*bx*)^ + *ce*^(−*dx*)^, with an *R*^2^ of 0.89. The function gives values for y_0_, the fully adapted firing rate, as 113 ± 1 AP s^−1^; an initial time constant for the first phasic response as 7 ± 0.9 ms; and a second time constant for the more slowly decaying part of the response as 102 ± 7.9 ms (Fig. 3c, red line).

### Response to changes in pulse interval

The effect of changes in pulse interval was tested with different trains of chirps consisting of four 20 ms pulses separated by pulse intervals that were varied in 5 ms steps from 5 to 60 ms. The chirp interval was kept at 140 ms. Each chirp pattern was presented twelve times sequentially and different chirp types were presented in pseudo-random order so that sequences with short pulse intervals were followed by chirps with long pulse intervals. Trains of 12 chirps with particular pulse interval characteristics were presented with a 2-s interval between stimulus types. The first response to a particular stimulus was excluded from analysis as its response was much greater than to subsequent stimuli in the train.

**Fig. 4:**
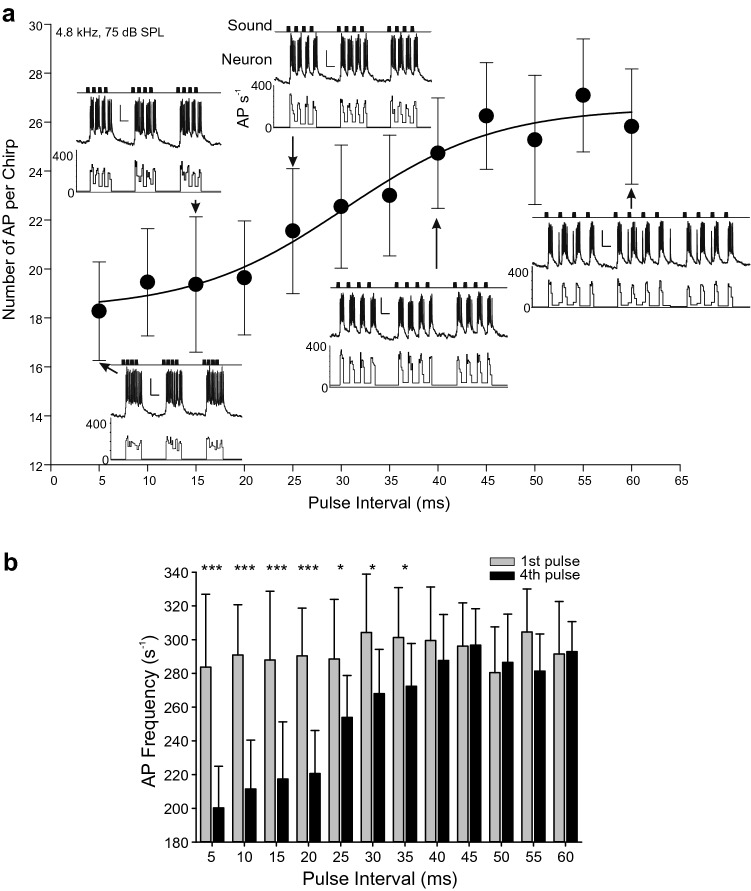
B-DARN1 response to altered pulse intervals. Presentation of four-pulse chirps with a constant pulse duration of 20 ms but with pulse intervals varied from 5 to 60 ms. **a** Mean total number of AP (± SD) evoked per chirp by chirps with different pulse intervals. Insets show representative responses of B-DARN1 to chirps of different pulse interval. Diagrams show the sound stimulus (top) the neuronal responses (middle) and the instantaneous AP firing rates (bottom). The scale bars are 10 mV and 50 ms in each case. **b** The peak instantaneous AP frequency following the first (gray bars) and fourth (black bars) pulses of chirps in which the pulse interval had been varied. Data are means ± SD, *N* = 11. Asterisks indicate significances of comparisons: *P < 0.05, ***P < 0.001. Data are from recording 1

When the pulse interval was 5 ms, the neuronal response to individual sound pulses merged into a single burst of AP surmounting a single sustained depolarization (Fig. 4a). With a pulse interval of 60 ms, the neuron almost completely repolarized between sound pulses and each response to a pulse was sharply defined by well separated bursts of AP surmounting a sustained depolarization (Fig. 4a). Songs with intermediate pulse intervals were characterized by a partial repolarization between bursts (e.g., 15 and 25 ms, Fig. 4a, and Fig. 2), with a spiking response to each individual sound pulse occurring.

The pulse interval affected the total number of AP evoked by a chirp even though the overall duration of sound was a constant 80 ms in each chirp type, likely due to a recovery from adaptation occurring over the time course of a chirp (graph Fig. 4a; Kruskal–Wallis test, $\chi^2_{11}$  = 107.1, *P* = 3.2 × 10^–18^, *N* = 11). The number of AP per chirp was 18.3 ± 1.6 AP when the pulse interval was 5 ms, and 32.5% lower than the 27.1 ± 1.2 AP evoked when the pulse interval was 55 ms. Numbers of AP were similar for pulse intervals from 10 to 20 ms and then increased near linearly for pulse intervals between 25 and 45 ms, before reaching a saturated response for pulse intervals greater than 45 ms (Fig. 4a).

Adaptation over the course of a chirp was analyzed by comparing the peak instantaneous AP frequency evoked after the first and fourth pulses for different pulse intervals (Fig. 4b). The peak AP frequency of the first pulse did not show any significant variation, indicating a constant level of activity following the consistent inter-chirp interval of 140 ms (Fig. 4b gray bars; average 293.3 ± 31.9 AP s^−1^; ANOVA, *F*_11, 120_ = 0.652, *P* = 0.781). The peak AP frequency in response to the fourth pulse, however, increased significantly with the pulse interval (Fig. 4b black bars; ANOVA, *F*_11, 120_ = 20.677, *P* = 1.3 × 10^–22^). Post hoc paired *t*-tests indicated that the peak AP frequencies of the fourth pulses were all significantly less than those of first pulses for pulse intervals < 40 ms, but for larger pulse intervals, there were no significant differences (Table 2). In response to the fourth sound pulse, the minimum peak instantaneous AP frequencies occurred for the 5 ms pulse interval chirps (200.3 ± 24.7 AP s^−1^); the peak firing rate progressively increased as the interval increased until it approached about 290 AP s^−1^, which was similar to the firing rate in response to the first pulse in a chirp.

**Table 2:** Results of paired *t-*tests comparing the highest instantaneous firing rate of B-DARN1 during the first and fourth pulse of chirps in which the pulse duration was held at a constant 20 ms, but the pulse interval was varied from 5 to 60 ms

Pulse interval (ms)	*t*_*10*_	*P*
5	4.87	6.5 × 10^–4^
10	11.98	3.0 × 10^–7^
15	6.88	4 × 10^–5^
20	12.19	2.5 × 10^–7^
25	3.0	0.013
30	2.5	0.032
35	2.57	0.028
40	0.79	0.448
45	0.05	0.980
50	0.48	0.640
55	2.03	0.070
60	0.18	0.860

### Response to attractive and unattractive chirp patterns

**Fig. 5:**
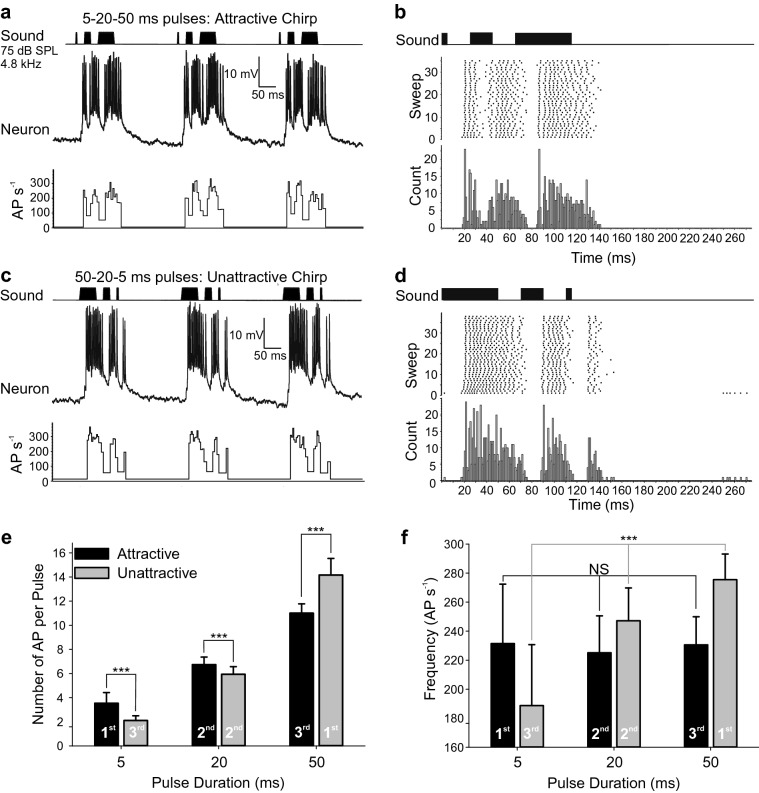
Response of B-DARN1 to attractive and unattractive chirp patterns. **a** Response to three attractive chirps with pulse durations of 5, 20 and 50 ms separated by 20 ms pulse intervals. Sound pattern (top), neuronal response (middle) and instantaneous AP firing rates (bottom). **b** Raster plot and PST histogram of B-DARN1 response to the 5–20–50 ms stimulus. **c** Response of B-DARN1 to three successive unattractive chirps with reversed pulse durations of 50, 20 and 5 ms separated by 20 ms pulse intervals. **d** Raster plot and PST histogram of B-DARN1 spike activity to the 50–20–5 ms stimulus. **e** Mean number of AP (± SD) evoked by the 5, 20, and 50 ms sound pulses when presented in the attractive (black bars) and unattractive (gray bars) order, *N* = 16. **f** Mean firing rates (± SD) to the different pulse lengths when presented in the attractive (black) and unattractive (gray) order. NS indicates not significant; *** indicates *P* < 0.001 in Mann–Whitney paired comparisons (**e**) and Kruskal–Wallis tests comparing firing rates across all three pulses (**f**). Data are from recording 1

Although natural songs show little variation from the 40 ms pulse-period pattern, female *G. bimaculatus* will show clear phonotaxis to some artificial song patterns, particularly if the first pulse is unnaturally short, whereas the length of the final pulse in a chirp is less critical (Hedwig and Sarmiento-Ponce, 2017). Conversely, reversing these artificial patterns so that chirps lead with a long pulse and end with a short pulse generates unattractive songs with significantly reduced phonotaxis. The response of B-DARN1 to attractive chirps consisting of 5, 20 and 50 ms pulses (Fig. 5a, b) was compared to its response to an unattractive pattern with 50, 20 and 5 ms pulses (Fig. 5c, d). The pulse intervals were 20 ms and the chirp interval was 185 ms (chirp rate 3.33 s^−1^).

As expected from the phaso-tonic responses to songs with different pulse durations (Fig. 3), the longer pulses within these attractive and unattractive chirps evoked larger numbers of AP (Fig. 5e), but the short pulses were over-represented in the spiking activity. The average duration of spiking activity lasted for 14.3 ± 4.4 ms for the 5 ms pulses (2.86 × longer than the duration of the stimulus), 27.6 ± 4.6 ms for the 20 ms pulses (1.38 × longer than stimulation duration) and 52.2 ± 3.7 ms for the 50 ms pulses (1.04 × longer than stimulus duration). The mean number of AP increased from 3.0 ± 1.2 for 5 ms stimuli, through to 6.4 ± 0.8 for 20 ms stimuli and 13.5 ± 2.1 for 50 ms stimuli; so, while the pulse duration increased tenfold, the number of AP only increased by a factor of 4.5 (Fig. 5e).

The order of presentation also affected the number and frequency of AP elicited by each pulse, with evidence of adaptation occurring over the course of a chirp (Fig. 5e, f). In the attractive chirps, the leading 5 ms pulse evoked 3.9 ± 1.1 AP compared to only 2.2 ± 0.4 AP evoked by the 5 ms in the unattractive chirp when it was in the last position (Mann–Whitney *U* test, *Z*_30_ = 4.66, *P* = 3 × 10^–6^). Similarly, the 50 ms pulse evoked more AP when it was in the leading position in the unattractive chirp (15.1 ± 1.2) compared to when it was last in the attractive chirp (11.4 ± 0.7 AP; Mann–Whitney, Z_30_ = 4.92, *P* = 1 × 10^–6^). Also, when the central 20 ms pulse was preceded by the long 50 ms pulse in the unattractive pulse, there was a significant decrease in the number of evoked AP (6.0 ± 0.5) compared to the number of AP in the attractive pulse (6.9 ± 0.7 AP; Mann–Whitney, *Z*_30_ = 3.31, *P* = 9.3 × 10^–4^). This effect led to a remarkable difference in average AP firing rate across the different chirp types (Fig. 5f): in the attractive chirps the mean firing rate was very similar across all three pulses (means 231 ± 41, 225 ± 25 and 231 ± 19 AP s^−1^; Kruskal–Wallis test, $\chi^2$  = 0.23, *P* = 0.891), whereas for the unattractive chirp the firing rate over the course of the chirp decreased progressively with each sound pulse by a total of 31% (275 ± 18, 247 ± 23 and 189 ± 42 AP s^−1^; Kruskal–Wallis test, $\chi^2$ = 30.4, *P* = 1.3 × 10^–7^).

### Effect of sound direction

**Fig. 6:**
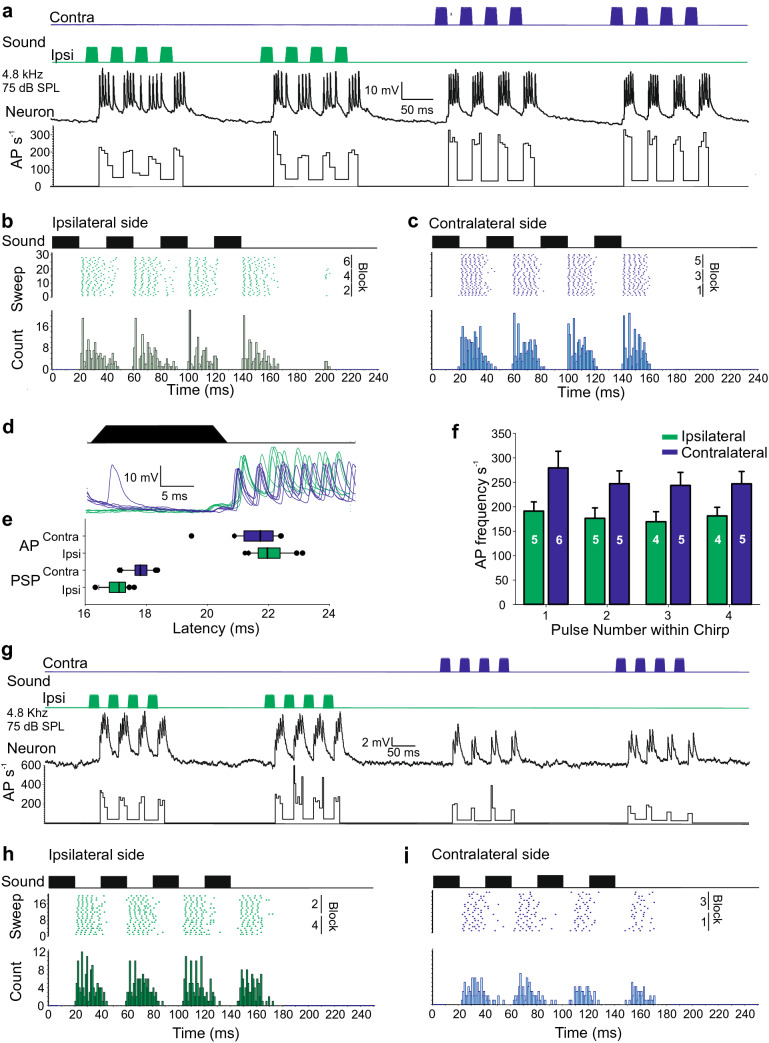
The effect of sound direction on the response of B-DARN1 Data in **a**–**f** from recording 1. **a** B-DARN1 activity (middle) and its instantaneous AP rate (bottom) when stimulated by chirps alternating between an ipsilateral (green) and contralateral (blue) direction every 10 chirps (top). The data show a transition from ipsilateral to contralateral stimulation. **b**, **c** Raster plot and PST histograms of B-DARN1 spike response when stimulated from the ipsilateral (**b**, green) or contralateral side (**c**, blue). All direction-specific responses are shown together; the numbers identify the blocks of 10 chirps played sequentially before swapping to the alternate side. **d** Five overlaid B-DARN1 responses to stimulation from ipsilateral (green) or contralateral (blue); traces are aligned to the start of the chirp. **e** Boxplots of the latency to the first PSP and first AP from the start of chirps when sound was presented from ipsilateral or contralateral sides. **f** Average firing rate (± SD) of B-DARN1 per pulse depending on the direction of the sound stimulus, white numbers indicate the median number of AP per pulse. **g–i** Response of B-DARN1 to monaural stimulation in recording 2. The responses characteristics shown here differed from those characterized in parts **a**–**f**, by showing a stronger response to stimuli coming from the side ipsilateral to the neuron. **g** A transition from ipsilateral (left, green) to contralateral stimulation (right, blue). The bottom trace shows the instantaneous AP frequency. **h**, **i** Raster plot and PST histograms of the spike response when stimulated from the ipsilateral (**h**, green) or contralateral side (**i**, blue)

In the previous tests during recording 1, B-DARN1 had been stimulated binaurally by two simultaneously active speakers positioned 36° relative to the longitudinal axis of the cricket but activating each speaker alternately revealed strong direction-dependent responses to natural chirp patterns (Fig. 6). The active speaker changed every 10 chirps (forming a series of alternating blocks, Fig. 6a–c, g–i). There were contrasting responses in different recording of B-DARN1, which are difficult to reconcile. In recording 1, sound from the ipsilateral side caused lower AP firing rates, as shown by the instantaneous firing rate (Fig. 6a lowest trace) and the average firing rate to each sound pulse of a chirp (Fig. 6f). On average, one more AP per sound pulse was evoked when presented by the right/contralateral speaker (5.5 ± 0.8) than from the ipsilateral/left speaker (4.5 ± 0.6; *t*_158_ = 8.67, *P* = 5 × 10^–15^), and there was also a difference in the duration of the burst of AP (21.8 ± 3.0 ms when stimulated contralateral and 25.4 ± 3.7 ms when ipsilateral; *t*_158_ = 1.41, *P* = 5 × 10^–10^; Fig. 6a). The B-DARN1 activity in recording 1 is demonstrated in the collated raster plots and histograms for sound coming from either side (Fig. 6b, c). They reveal a 29% difference in the mean firing rate depending on the sound direction (Fig. 6f; 254.3 ± 31.3 AP s^−1^ when contralateral; 179.6 ± 20.7 AP s^−1^ when ipsilateral; ANOVA, effect of stimulus side, *F*_1, 152_ = 379.51, *P* = 5.2 × 10^–44^; effect of pulse number, *F*_3, 152_ = 10.83, *P* = 2.0 × 10^–6^). A post hoc comparison suggested that the first pulse in a chirp evoked a significantly higher AP frequency than subsequent pulses.

In recording 1, there was an initial PSP preceding the spiking response (see Fig. 2h). The latency to this first PSP differed significantly depending on the sound direction (Fig. 6d, e). The latency to ipsilateral presented pulses (17.0 ± 0.3 ms) was 0.8 ms shorter than the response to contralateral stimuli (17.8 ± 0.3 ms; *t*_38_ = 7.0, *P* = 4.1 × 10^–13^). The latency to the first AP when stimulated on the contralateral side was slightly shorter (21.6 ± 0.7 ms), than the latency, when stimulated on the ipsilateral side (22.0 ± 0.5 ms; *t*_38_ = 2.15, *P* = 0.038; Fig. 6d, e). The PSP latencies to unidirectional sound were both considerably shorter than when both loudspeakers were simultaneously active (20.1 ± 0.6 ms). As with the latency to PSP the latency to spiking was longer when both loudspeakers were active (22.8 ± 0.9 ms).

In the other two recordings (2 and 3), a different and contrasting response to monaural stimulation was seen (Fig. 6g–i), even though their structure indicated that the neuron was B-DARN1. In these recordings, the response to ipsilateral auditory stimulation was clearly stronger than when stimulated on the contralateral side. In recording 2 (Fig. 6g), there were 77.8% more AP per pulse when stimulated ipsilaterally, compared to contralaterally (ANOVA, *F*_1, 156_ = 555.97, *P* = 2.7 × 10^–53^; effect of pulse number *F*_3, 156_ = 25.29, *P* = 2.2 × 10^–13^; Median number of AP per pulse, 5, 5, 5, 4 for the ipsilateral side and 3, 3, 2, 2 for contralateral side; Fig. 6h, i). The average firing rate of the neuron was 54.6% higher when stimulated on the ipsilateral side (excluding the 10% of contralateral responses where only a single AP was evoked; (ANOVA, *F*_1, 144_ = 379.75, *P* = 3.3 × 10^–42^; effect of pulse number *F*_3, 144_ = 12.15, *P* = 3.9 × 10^–7^). There was also a briefer latency of response when stimulated on the ipsilateral side (21.7 ± 0.9 ms compared to 25.6 ± 2.3 ms on the contralateral side; *t*_23.32_ = 6.525, *P* = 1.1 × 10^–6^).

### Effect of high-frequency sound stimulation

**Fig. 7:**
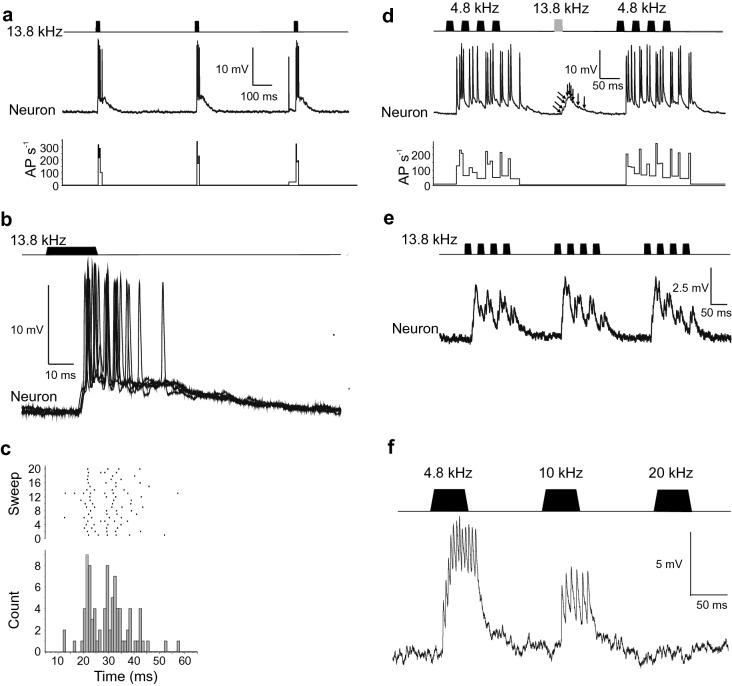
B-DARN1 response to high-frequency sounds. **a** Three 20 ms sound pulses at 13.8 kHz repeated at 500 ms intervals at 75 dB SPL (top), B-DARN1 responses (middle) and its instantaneous firing rate (bottom) in recording 1. **b** Five superimposed successive responses of B-DARN1 to 20 ms 13.8 kHz pulses aligned on the start of each sound pulse. **c** Raster plot and PST histograms of the B-DARN1 responses to the stimulus, aligned with the sweeps shown in **b**. **d** Response of B-DARN1 to a 20 ms 13.8 kHz pulse intercalated between 10 chirps played at 4.8 kHz at 75 dB SPL. The response to the high-frequency stimuli remained sub-threshold and revealed the input of PSPs (arrows). **e** Response of B-DARN1 to a chirp pattern with four 20 ms sound pulses and 20 ms inter-pulse intervals played at a high frequency of 13.8 kHz (at 75 dB SPL). **f** Response of B-DARN1 in recording 2:50 ms pulses with escalating frequencies of 4.8, 10 and 20 kHz at a sound intensity of 75 dB SPL; the incidence of AP was decreased at 10 kHz and the neuron failed to respond to 20 kHz sounds

When the cricket was stimulated at 500 ms intervals by brief 20 ms pulses of 13.8 kHz and 75 dB SPL, long-lasting (109.0 ± 14.6 ms) compound PSPs were evoked after a latency of 14.6 ± 0.6 ms in recording 1 (Fig. 7a, b). The PSPs were surmounted by a brief burst of 3.8 ± 0.6 AP (Fig. 7c), with an average frequency of 175.1 ± 21.2 AP s^−1^. Intercalating such high-frequency pulses within a train of ‘normal’ chirps at 4.8 kHz at an incidence of one high-frequency pulse per ten calling song chirps, abolished the spiking response to the high-frequency sounds, and revealed a barrage of individual PSPs producing a compound response (indicated by arrows in Fig. 7d), whilst leaving the response to the chirps unaltered (Fig. 7d). The high frequency of inputs and short latency of the graded response suggests that they come from AN2, an ascending interneuron that is more strongly tuned to higher frequencies (Wohlers and Huber 1982). Playing chirp-like patterns (chirps with four pulses of 20 ms with 20 ms pulse intervals and 140 ms chirp intervals) at 13.8 kHz also did not evoke AP in B-DARN1, but instead compound PSPs which followed the pulse pattern of the chirps (Fig. 7e). Again, the structure of individual PSPs forming this compound response could be discerned.

In recording 2 of B-DARN1, the neuron was presented with a sequence of three 50 ms pulses with a sound intensity of 75 dB SPL and escalating frequency, 4.8, 10 and 20 kHz at 110 ms intervals (Fig. 7f). The number of evoked APs decreased with increasing carrier frequency, from a peak mean burst frequency of 198 ± 26 AP s^−1^ at 4.8 kHz, to 140 ± 15 AP s^−1^ at 10 kHz and the neuron failed to respond at all to 20 kHz pulses (*N* = 12). The latencies were 19.3 ± 1.8 ms for the 4.8 kHz stimuli and 29.9 ± 1 ms for the 10 kHz stimuli in this recording. The B-DARN1 in recording 3 responded in a near identical manner to these same three frequencies (data not shown).

**Fig. 8:**
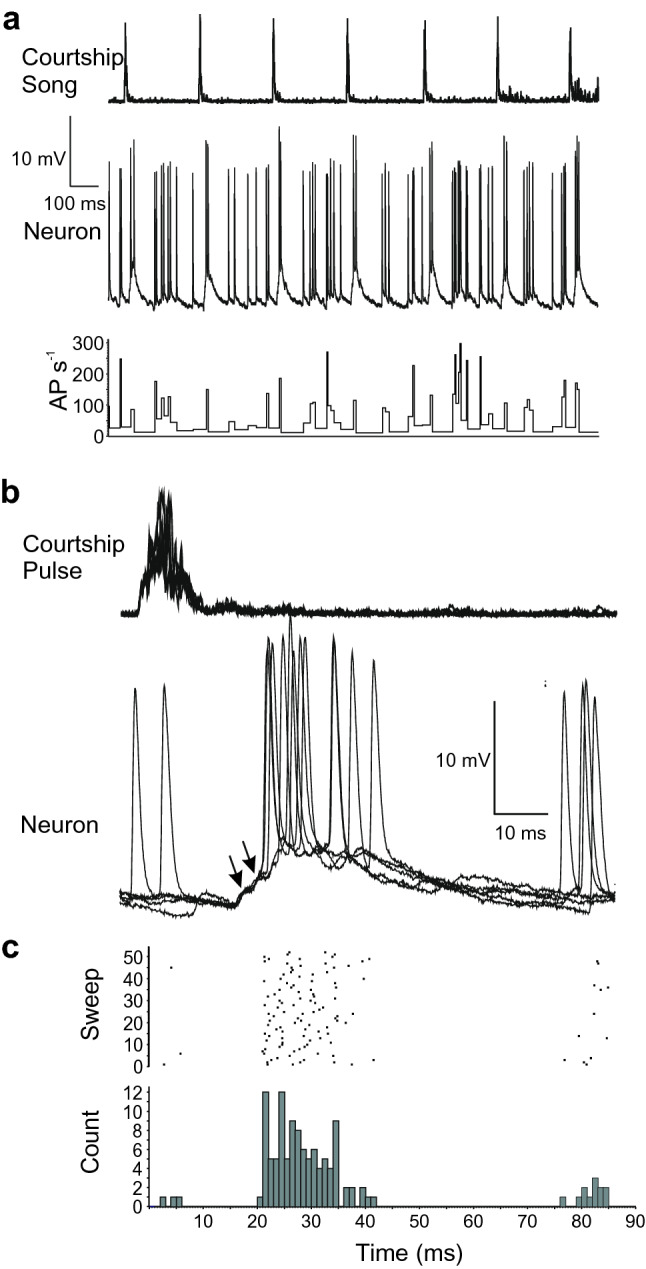
**a** Presentation of courtship song (top) and response of B-DARN1 (middle) and its instantaneous AP frequency (bottom) in recording 1. Each high-frequency courtship song pulse evoked a PSP surmounted by two AP against a background of increased background firing. **b** Overlay of five successive responses of B-DARN1 to courtship song pulses, showing the summating PSPs (arrows) that lead to spiking activity. **c** Raster plot and histogram showing the spiking response of B-DARN1 to courtship song, triggered by the start of the courtship song pulse. The histogram is aligned with the sweeps shown in **b**

Courtship song in *G. bimaculatus* consists of 12–15 ms long sound pulses repeated at ~ 3.75 Hz and is dominated by frequencies of 12–16 kHz intercalated with a series of low-amplitude 4.6–4.9 kHz sound pulses (Libersat et al. 1994; Lin and Hedwig 2021). B-DARN1 was presented with courtship song in recording 1; it responded with an elevated rate of ‘background’ AP firing (approximately 10–20 AP s^−1^) likely tied to the low-amplitude pulses of the song (Fig. 8a); while each high-frequency sound pulse evoked a high amplitude long-lasting compound PSP (39.2 ± 9.1 ms; Fig. 8b) surmounted by 2.1 ± 0.4 AP reaching frequencies of 143 ± 46 AP s^−1^, which was aligned with the onset of the high-frequency courtship song pulse (Fig. 8c). The rising phase of the compound PSP was composed of at least two individual PSPs before the spiking threshold was reached (Fig. 8b).

## Discussion

Using intracellular recordings in the vicinity of the auditory brain neuropil in *G. bimaculatus*, we revealed the structure and response properties of a descending auditory-responsive interneuron, B-DARN1. Its characteristics clearly stand out from the structure and response properties of previously described descending auditory interneurons, and it considerably adds our understanding of the organization of the cricket auditory pathway. The neuron could be part of a fast descending pathway, supporting auditory motor responses.

B-DARN1 responds robustly to the calling song frequency of 4.8 kHz with high spiking activity, indicating a connection with AN1, but also shows evidence of predominately sub-threshold inputs from the high-frequency AN2 pathway. The high spiking rates and rapid latency suggest this neuron is at least partially independent of the pattern recognition network of local brain interneurons, which produces a sparse output.

### Structure of B-DARN1

A ring-like neuropil in the ventral protocerebrum in the brain of *G. bimaculatus* is essential for recognizing the pattern of calling song. Not only does it contain the axonal arborization of the ascending, calling song frequency-sensitive auditory interneuron, AN1, but several local auditory interneurons that have a critical role in pattern recognition also have their arborizations confined to this neuropil region. (Kostarakos and Hedwig 2012, 2017; Schöneich et al. 2015). The second ascending auditory interneuron, AN2, which is most strongly responsive to auditory frequencies higher than calling song, also has an extensive arborization within the ring neuropil. The arbor of B-DARN1 also falls entirely within the ring-like neuropil, and this by itself suggests a strong link to auditory processing. While two recordings (2,3) were made at the transition between the axon and the main neurites in the ring, the electrode location during the longest recording (1) was well outside this auditory neuropil, it is unlikely that the records conflate the response of a local auditory interneuron with an inadvertent concomitant stain of AN1. Two of the three stains come with some evidence for two cell bodies being labeled (Fig. 1), indicating that B-DARN1 neurons may come as a pair of sibling neurons or that there may be a group of B-DARN neurons. Other descending auditory-responsive interneurons lack the close proximity to the ring-like auditory neuropil (Boyan and Williams 1981; Böhm and Schildberger 1992; Brodfuehrer and Hoy 1990; Staudacher 2001; Staudacher and Schildberger 1998, Zorovic and Hedwig 2013), indicating that these neurons may be activated at a later stage of auditory processing. As the axonal projection of B-DARN1 has not yet been revealed, it is unknown which ganglia of the nerve cord are supplied with its activity.

### Synaptic input to B-DARN1

**Fig. 9:**
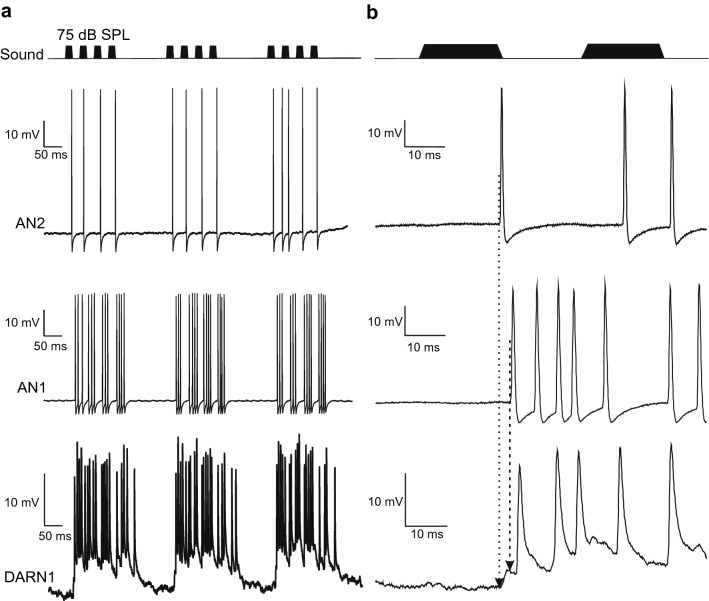
The response of B-DARN1 (bottom) compared to the ascending auditory interneurons AN2 (top) and AN1 (middle) to calling song at 4.8 kHz and 75 dB SPL. The data are from three different recordings in different crickets (B-DARN1 data from recording 1). **a** Response to three successive chirps of calling song. **b** Expanded view of the response to the first two pulses of a chirp, with arrows indicating the latencies of AN2 and AN1 relative to the response of B-DARN1

In field crickets, two prominent interneurons provide the brain with auditory activity, AN1 narrowly tuned to the carrier frequency of the calling song and AN2, which is broadly tuned and more responsive to high-frequency signals like bat sonar calls or the males’ courtship song in field crickets like *G. bimaculatus* and *G. campestris* (Hardt and Watson 1994; Hennig 1988; Moiseff and Hoy 1983; Rheinlaender et al. 1976; Schildberger 1984; Wohlers and Huber 1982). This may form the basis for categorical frequency responses like attraction to calling song and avoidance of bat-like signals (Wyttenbach et al. 1996). The synaptic input to B-DARN1 reveals strong spiking responses to sound pulses at the calling song carrier frequency (Fig. 2h) but the neuron also responded to high-frequency signals (Figs. 7, 8), albeit generally more weakly and in many cases only PSPs were evoked (Fig. 7d, e), indicating it may receive its input from both AN1 and AN2. The synaptic and spiking response, however, was stronger for the low frequency sound pulses. In *G. bimaculatus* B-DARN1 send auditory activity related to calling and courtship song with short latency back to posterior ganglia. The short latency of the synaptic response was very close to the latency of AN1 spike activity measured in the brain (Schildberger 1984; Schöneich et al. 2015). During sustained stimulation with calling song AN2 will start to respond with a single AP to each pulse of the song (Fig. 9a), which reaches the brain after a shorter latency than AN1 (Fig. 9b), (Boyan and Williams, 1982). It is, therefore, possible that AN2 provides the first PSP in the auditory response of B-DARN1, priming the neuron for the subsequent input from AN1 (Fig. 9).

### Response to calling song patterns

The short latency of the B-DARN1 response may not leave much time for complex auditory processing. In recording 2 B-DARN1 spike activity matched response patterns of AN1 which copies the pulse pattern (Wohlers and Huber 1982; Kostarakos and Hedwig 2012) (Fig. 2e); however in recording 1, its activity was less precise in representing the pulse pattern as sound pulses elicited a bimodal response (Fig. 2b, d), the origin of this is not obvious. The bimodal pattern disappeared when DARN was stimulated monoaurally (Fig. 6 a–f) and returned when binaural stimulation was resumed. The disparity between the change in PSP latency (earlier on ipsilateral side compared to contralateral stimulation) and the onset of spiking (later on the ipsilateral side), (Fig. 6d, e) during monaural stimulation may indicate the presence of a binaural input into B-DARN1. A feature of B-DARN1 was the strong, 29% difference, in AP firing rate between ipsilateral and contralateral stimulation during recording 1.

Two contrasting patterns of response to monaural response were seen in different recordings. In recording 1 both ipsi- and contralateral stimulation elicited a vigorous response, and even though similar numbers of AP were evoked by stimuli from both sides, the firing rate was highest when stimulated by contralateral stimuli (Fig. 6a–f). In the other two recordings (2,3), the response was clearly substantially stronger to ipsilateral stimuli (Fig. 6g–i). It is unclear what underlies these conflicting response properties: there could be a population of B-DARN neurons, which though anatomically very similar exhibit different physiological properties; it is possible that B-DARN1 has different modulatory states in which the ipsilateral and contralateral inputs can have different synaptic weights depending upon circumstances; or the sound fields around the recorded specimens may have had an impact, distorting directional information.

In response to long sound pulses, the phasic onset of the B-DARN1 spike activity during the first 20 ms stood out, similar to the phasic onset activity of auditory afferents and thoracic local neurons (Nabatiyan et al. 2003). Compared to AN1, whose adaptation curve during sustained 500 ms stimuli can be adequately described by a simple exponential decay function with a time constant of approximately 40 ms (Benda and Hennig 2008), B-DARN1 appears to undergo a more rapid initial adaptation with a briefer time constant of only 7 ms, followed by a period of slower adaptation with a time constant of approximately 100 ms. The fully adapted tonic firing rates of both B-DARN1 and AN1, however, appear to be similar at 113 and ~ 100 AP s^−1^, respectively (Benda and Hennig, 2007).

While local auditory brain neurons show a pronounced tuning to the temporal features of the calling song (Kostarakos and Hedwig 2012), the stimulus response curves of B-DARN1 resemble the tuning of AN1 to such stimuli, which copies the auditory pulse pattern without a particular preference (Wohlers and Huber 1982; Kostarakos and Hedwig 2012). The activity of B-DARN1 therefore is unlikely to be driven by the output activity of the pattern recognition circuit in the brain—at least under the given recording situation. Indeed, the short latency of response of B-DARN1 precludes the involvement of the delay-line and coincidence detection circuit, which for pattern recognition requires a processing time corresponding to the pulse period (~ 40 ms). We did not find any evidence that the response of B-DARN1 evolved over the course of a chirp or over longer periods of stimulation.

Female *G. bimaculatus* respond well to chirp patterns with pulses of increasing duration; however, these chirps elicit a significantly lower phonotactic response when played in reverse pulse order (Hedwig and Sarmiento-Ponce 2017). A link to the different responses to these attractive and unattractive chirps may be seen in the response of B-DARN1, as its maximum spike frequency in response to pulses of different length was stable for the attractive pattern, but not so for the unattractive pattern (Fig. 5f). This difference, however, may be related to adaptation processes in the auditory pathway, rather than to pattern-specific processing in the brain.

### Possible functions of B-DARN1 in cricket phonotaxis

It is surprising to find an auditory brain neuron that appears to simply reflect the ascending activity back towards the posterior CNS. Functions that may be assigned to B-DARN1 could well be covered by the thoracic auditory neurons. We can only provide tentative assumptions, which relate to open questions of auditory-to-motor integration in phonotactic behavior.

The organization of the cricket ascending auditory pathway and the localization of the pattern recognition circuit in the brain, require some descending interneurons that forward the output of the pattern recognition process to the thoracic ganglia. These neurons have not yet been identified. Compared to some descending neurons which require high sound intensities and show rather weak auditory responses (Staudacher and Schildberger 1998; Staudacher 2001, Zorovic and Hedwig 2013), B-DARN1 comes with a strong and reliable response to calling song sound pulses, even at low sound intensities sufficient for phonotactic behavior. B-DARN1, therefore, could provide auditory information to posterior ganglia, which are bypassed by the ascending interneurons, but may contribute to the control of auditory behavior, like neurons with auditory responses in the locust suboesophageal ganglion (Boyan and Altman 1985).

B-DARN1 showed a clear, albeit variable, directional response, with contralateral stimuli in some recordings eliciting a stronger response, which is surprising as AN1 spike activity would be lower in a corresponding stimulus situation (Wohlers and Huber 1982). The B-DARN1 latency to ipsilateral stimuli was, however, shorter (Fig. 6b-f). During phonotaxis crickets move their antennae, and the prothoracic segment towards the side of acoustic stimulation (Witney and Hedwig 2011; Petrou and Webb 2012; Ntelezos 2022). Interneurons controlling antennal movements (Horseman et al. 1997) and motoneurons controlling neck muscles (Honegger et al. 1984) are located in the suboesophageal ganglion. The ascending interneurons AN1 and AN2 do not provide auditory information to the suboesophageal ganglion. B-DARN1 may supply the suboesophageal ganglion with auditory activity to support the sound evoked responses of the antennal and neck motoneurons.

During phonotactic walking, crickets rapidly adjust the trajectories of their legs to the direction of acoustic stimulation (Witney and Hedwig 2011; Petrou and Webb 2012). Although the relevant information and activity may be provided at the thoracic level by prothoracic auditory interneurons (Wohlers and Huber 1982; Stiedl et al. 1997) B-DARN1 may play an additional role in forwarding auditory information to the thoracic system due to its short latency responses. Correct pattern recognition is undoubtedly needed to initiate and maintain phonotaxis in the long term, but once committed to performing phonotaxis, crickets will respond and turn towards incorrect sound pulses or partial chirp patterns intercalated into song trains. It takes several seconds of being persistently presented with consistently wrong sound patterns for phonotaxis to cease entirely (Hedwig and Poulet, 2004; 2005; Poulet and Hedwig 2005). Presumably, an excitatory drive that is calling song frequency specific,—but not pattern-specific—is responsible for sustaining short term phonotactic motor responses, which may assist in maintaining a track towards a calling cricket under conditions where local variations in the sound field or ambient noise occasionally distort calling song. It is possible that non pattern-specific descending neurons such as B-DARN1 could contribute to this excitatory drive.

The activity of B-DARN1 even opens the possibility of another delay-line circuit in the auditory system of crickets. The near 20 ms latency of response in B-DARN1 may be of significance; if this was coupled with a similar conduction time back down to the thoracic ganglia it would introduce a near 40 ms delay, coinciding with the pulse frequency of calling song, setting up a delay-line and coincidence system based on conduction and processing delays to boost auditory responses for particular temporal patterns as outlined by Reiss (1964). In the ascending pathway the ~ 20 ms latency arises from transduction processes, sensory axon conduction, a synaptic delay, and then further conduction in AN1. The latency in the descending pathway would depend on conduction velocities within B-DARN1 (which appears to have a smaller and therefore slower axon than AN1 or AN2 as it is not discernible in extracellular connective recordings (e.g., Benda and Hennig 2008)); the number of neurons through which the signals pass, and the synaptic processes governing them, for which we lack any information.

### Response to high-frequency sounds

High-frequency sounds elicit bat avoidance behavior in flying crickets, a behavior that requires pathways to the brain be intact (Nolen and Hoy 1984). Ultrasound-sensitive local and descending brain neurons occur in *Teleogryllus oceanicus* (Brodfuehrer and Hoy 1990); however, the neurons that control the avoidance steering have not been identified. With its short latency responses to high-frequency signals, B-DARN1 might contribute to the bat avoidance steering of flying crickets.

B-DARN1 showed clear synaptic and spiking response to high-frequency sound pulses, the spiking activity, however, was lower than to calling song pulses, and the response became sub-threshold when embedded in a sequence of calling song chirps (Fig. 7d). Its response to courtship song pulses revealed a pronounced synaptic input, which likely was driven by the activity of AN2 and could support responses during courtship behavior.

## Conclusion

The structure and response properties of B-DARN1 offer new insight into the organization and function of the cricket auditory pathway and will support future neurophysiological approaches to reveal underlying mechanisms of auditory processing.

## Data Availability

Details for data can be obtained from the authors.

## Abbreviations

B-DARN1: Brain descending auditory-responsive neuron
LN: Local neuron
AP: Action potential
PSP: Post-synaptic potential
AN1, AN2: Ascending auditory neurons
